# Widespread anti-CRISPR proteins in virulent bacteriophages inhibit a range of Cas9 proteins

**DOI:** 10.1038/s41467-018-05092-w

**Published:** 2018-07-25

**Authors:** Alexander P. Hynes, Geneviève M. Rousseau, Daniel Agudelo, Adeline Goulet, Beatrice Amigues, Jeremy Loehr, Dennis A. Romero, Christophe Fremaux, Philippe Horvath, Yannick Doyon, Christian Cambillau, Sylvain Moineau

**Affiliations:** 1 Département de biochimie, de microbiologie, et de bioinformatique, Faculté des sciences et de génie, Groupe de recherche en écologie buccale, Faculté de médecine dentaire, Université Laval, Québec City, QC G1V 0A6 Canada; 2 Farncombe Family Digestive Health Research Institute, McMaster University. Department of Medicine, Faculty of Health Sciences, McMaster University, Hamilton, ON L8S 4K1 Canada; 3 Centre Hospitalier Universitaire de Québec Research Center, Université Laval, Québec City, QC G1V 4G2 Canada; 4 Architecture et Fonction des Macromolécules Biologiques, Aix-Marseille Université, Campus de Luminy, Case 932, 13288 Marseille Cedex 09, France; 5 Architecture et Fonction des Macromolécules Biologiques, Centre National de la Recherche Scientifique (CNRS), Campus de Luminy, Case 932, 13288 Marseille Cedex 09, France; 6 DuPont Nutrition and Health, 3329 Agriculture Dr, Madison, WI 53716 USA; 7 DuPont Nutrition and Health, BP 10, 86220 Dangé-Saint-Romain, France; 8 Félix d’Hérelle Reference Center for Bacterial Viruses, Faculté de médecine dentaire, Université Laval, Québec City, QC G1V 0A6 Canada

## Abstract

CRISPR-Cas systems are bacterial anti-viral systems, and bacterial viruses (bacteriophages, phages) can carry anti-CRISPR (Acr) proteins to evade that immunity. Acrs can also fine-tune the activity of CRISPR-based genome-editing tools. While Acrs are prevalent in phages capable of lying dormant in a CRISPR-carrying host, their orthologs have been observed only infrequently in virulent phages. Here we identify AcrIIA6, an Acr encoded in 33% of virulent *Streptococcus thermophilus* phage genomes. The X-ray structure of AcrIIA6 displays some features unique to this Acr family. We compare the activity of AcrIIA6 to those of other Acrs, including AcrIIA5 (also from *S. thermophilus* phages), and characterize their effectiveness against a range of CRISPR-Cas systems. Finally, we demonstrate that both Acr families from *S. thermophilus* phages inhibit Cas9-mediated genome editing of human cells.

## Abstract (web summary)

Some phages carry genes coding for anti-CRISPR (Acr) proteins that interfere with the activity of bacterial CRISPR-Cas systems. Here, Hynes et al. characterize a new Acr family from streptococcal phages and investigate its potential in genome-editing applications.

## Introduction

The rapid progression of CRISPR-Cas technology, from the discovery of its DNA cutting-activity^1^ to its widespread adoption as a genome-editing tool, has been astonishing. Its native function as a bacterial anti-viral system was uncovered in the dairy bacterium *Streptococcus thermophilus* a decade ago^2^. Upon exposure to strictly lytic streptococcal viruses (bacteriophages or phages), only a few bacterial cells would survive having incorporated a short 30 nucleotide (nt) sequence matching phage genomic DNA into the “memory” of this adaptive immune system: the CRISPR array^2^. This array is transcribed to form short RNAs named crRNAs, which serve to lead the Cas9 nuclease to its DNA cleavage target through RNA-DNA base-pairing, thereby providing CRISPR immunity^3^. For genome-editing applications, a synthetic version known as the single-guide RNA (sgRNA) can be designed to direct Cas9 to its target^4,5^. Even in the initial discovery of the anti-phage role of CRISPR-Cas systems in bacteria, it was evident that phages had means of bypassing that immunity—a phage with a single point mutation in the 30-nt sequence targeted by the crRNA^2^, or in the flanking proto-spacer adjacent motif (PAM)^6^ could freely infect the CRISPR-immunized bacterial host. This came as no surprise, as phages and bacteria are locked in an arms race as old as they are. What was surprising, given the ease with which phages bypass this immunity, was the discovery of phage proteins specifically interfering with CRISPR-Cas systems: anti-CRISPRs (Acrs)^7^.

The field of Acrs has rapidly garnered interest, largely due to potential applications modulating the cleavage activity of various Cas9 proteins. Tight control over Cas9 could prevent off-target cleavage in genome-editing applications^8^, or lock Cas9 into useful catalytically inactive states^9^. Bioinformatic methodologies have uncovered a number of Acrs that interfere with different types of CRISPR-Cas systems^10–13^ in a variety of manners^9,13–20^. To date, the in-depth characterization of eight Acrs (four against type I, four against type II CRISPR-Cas systems^21,22^) has revealed at least six distinct mechanisms of action^13,20,23^. Initially these Acrs were invariably found in temperate phages (phages that can enter a latent “prophage” state in the bacterial genome), prophage remnants, and horizontally acquired genomic islands^23^. In 2017, Acrs were discovered in the genomes of virulent phages—phages that cannot become prophages—infecting *Listeria monocytogenes*^24^ and *S. thermophilus*^25^. Even for the two Acrs detected in virulent phages (AcrIIA4 from *L. monocytogenes* and AcrIIA5 from *S. thermophilus*), the majority of their homologs were found in temperate phages^24,25^. While this is likely a reflection of the relative paucity of virulent phage sequences, it could also imply that the short-lived association of a virulent phage with its host would reduce the benefits of carrying an Acr. Virulent phages might also simply have distinct Acrs, given the different evolutionary pressures they face. Supporting this notion, the AcrIIA4 homologs in virulent *Listeria* phages share at most 41% ID with the reference from a prophage. Interestingly, the Acrs with orthologs in virulent phages are two (AcrIIA4 and AcrIIA5) of the only three (alongside AcrIIA2) to block the activity of the genome-editing tool SpCas9^4,26^, derived from the type II-A CRISPR-Cas system of *Streptococcus pyogenes*^22^. Of these three proteins with demonstrated inhibition of SpCas9, only the structure and mode of action of AcrIIA4 are known^8,27,28^. AcrIIA4 competes with the dsDNA target of the SpCas9-sgRNA complex through interaction with the PAM-binding domain^27,28^.

In this study, we take advantage of the CRISPR immunity in *S. thermophilus*^29^ and a large collection of virulent phages as hunting ground for new Acrs. We probe a dataset of 254 (largely virulent) *S. thermophilus* phage genomes, including new public genomic sequences^30^ as well as our set of phage genomes, to search for Acrs. We capitalize on a methodology^25^ derived from bacterial CRISPR-based immunization^31,32^, armed with the knowledge that activity against one of the two distinct *S. thermophilus* type II-A CRISPR-Cas systems (CR1/St1Cas9 and CR3/St3Cas9) can yield Acrs with activity against SpCas9^25^.

## Results

### Discovery of new Acrs

**Fig. 1:**
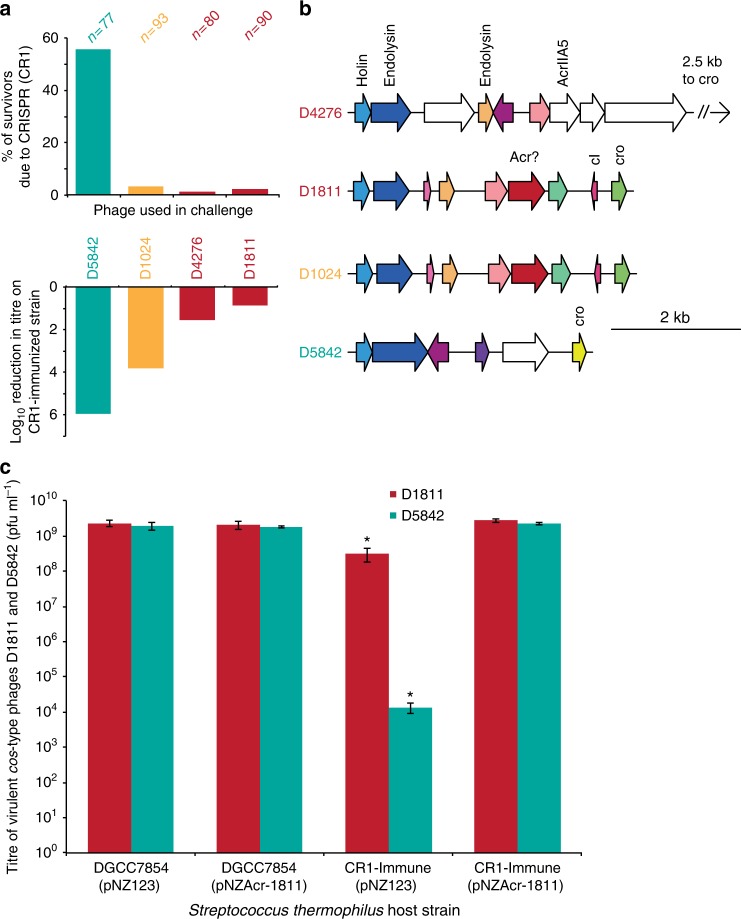
Discovery of a new Acr. **a** Profiles of immunity generation (top, where *n* = the number of survivors screened) and ability to bypass immunity (bottom) of CRISPR-sensitive (green), CRISPR-resistant (red), and CRISPR-intermediate (ochre) phages. Log reductions from three technical replicates. **b** Representation of the genomic region in which *acrIIA5* was found in D4276 for each phage in **a**. ORFs, represented by an arrow, are color-coded if their product shares >50% aa identity with another elsewhere in any of the other phages, and white if it does not. Functions are annotated for the first instance of any ortholog. “Acr?” was investigated for being downstream of a gene (pink) conserved in D4276. **c** Titres of a CRISPR-sensitive (green) or CRISPR-resistant (red) phage on their host or a CR1-immunized version thereof, in the presence or absence of the putative Acr. The presence of the Acr restores the titre of both phages to the levels observed in the absence of immunity. The titres are from three biological replicates, each of three technical replicates. Error bars represent standard deviation, and a star (*) indicates a significant difference from each other column in the graph, as calculated by one-way analysis of variance (ANOVA) and Tukey honest significant difference (HSD) test (*p* < 0.01)

AcrIIA5 was previously discovered in the virulent phage D4276, infecting *S. thermophilus* strain DGCC7854, after the phage was found to bypass CRISPR immunity against it^25^. We identified another virulent phage infecting the same strain, phage D1811. When strain DGCC7854 was challenged with phage D1811, only a small proportion of the surviving colonies had acquired a new spacer in their CRISPR arrays, in contrast to what is typically observed during CRISPR-based immunity (Fig. 1a). Genome analysis indicated that phage D1811 did not contain *acrIIA5* or a homolog thereof. Furthermore, when used to challenge a CR1-immunized strain targeting a 30-nt conserved phage genomic region flanked by a suitable PAM, phage D1811 was still able to form plaques and replicate efficiently—even better than phage D4276, which harbors *acrIIA5* (Fig. 1a). This led us to believe that the virulent *S. thermophilus* phage D1811 possessed an unidentified Acr.

We compared the genome organization of the two phages and observed that the *acrIIA5* gene in the genome of phage D4276 is located downstream of a conserved gene (Fig. 1b, pink), which is also present in D1811. As gene neighborhood analysis has been useful in other cases of Acr discovery^12^, we cloned the D1811 gene (D1811_026) located downstream of this conserved gene to check for anti-CRISPR activity. Expression of the putative *acr* on a plasmid in a CR1-immunized *S. thermophilus* strain completely abolished immunity against the CRISPR-sensitive virulent phage D5842 (Fig. 1c, green), and even eliminated the residual resistance observed against phage D1811 (Fig. 1c, red). This new Acr, which bears no similarity to any Acr characterized to date, was named AcrIIA6.

**Fig. 2:**
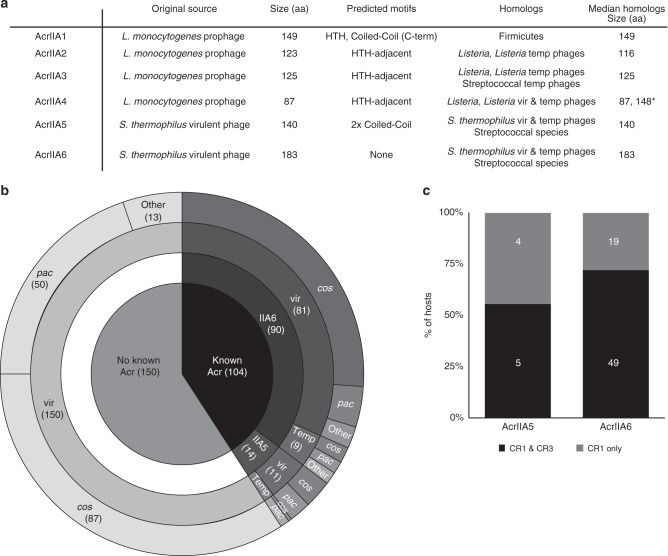
Bioinformatic analyses of type II-A Acrs. **a** A summary of key features of type II-A Acrs. For orthologs, virulent and temperate are abbreviated “vir” and “temp,” respectively. The star (*) indicates a bi-modal distribution in size, with a smaller set clustering around 87 aa and a larger set (present in virulent phages) clustering around 148 aa. **b** Categorizing 254 available *S. thermophilus* phage genomes for presence of a known Acr, by Acr family, phage type (vir = virulent, temp = temperate), and group (*cos*, *pac*, other). **c** A breakdown of AcrIIA5-containing and AcrIIA6-containing phages by CRISPR-Cas systems present in the host, where the host’s CRISPR types are known. The number of hosts for each category is presented in white over the bar graphs, and no host possesses only the CR3 system

AcrIIA6 proved difficult to characterize using standard bioinformatics tools—neither it, nor the predicted proteins whose genes are flanking *acrIIA6* had any recognizable motif, e.g., helix-turn-helix (HTH) or coil-coil motifs, previously associated with other Acrs (Fig. 2a). A distinguishing feature of the protein was its size. At 183 amino acid (aa) residues, it is the largest (+23%) known type II-A Acr. Interestingly, the few orthologs of AcrIIA4 found in virulent phages were ~70% larger (148 aa) than their counterparts in prophages, and were similarly not found adjacent to proteins with HTH motifs present for their prophage orthologs^24^. We used AcrIIA6 to probe *S. thermophilus* phage sequences for homologs of the protein. Remarkably, 90 out of the 254 (35.4%) phage genomes contained an *acrIIA6* ortholog (criterion = 75% nucleotide identity (ID) over 75% of the gene length). The same 90 sequences were returned using less stringent criteria; 40% ID over 40% of the gene length. Of note, 81 of these genes were found in the genome of 242 virulent *S. thermophilus* phages (33.5% prevalence) (Fig. 2b), while nine of them were found in 12 temperate phages (75% prevalence). By comparison, only 14 (5.5%) out of the 254 genomes contained an *acrIIA5* ortholog, and no phage genome contained both *acrIIA5* and *acrIIA6* (Fig. 2b). The prevalence of AcrIIA6 in virulent phages is lower than that of AcrIIA4 (16/23 public complete genomes). It is noteworthy that all 12 known *S. thermophilus* temperate phages contained either an *acrIIA5* (25%) or *acrIIA6* (75%). This may suggest a greater need for genetic elements that associate long-term with a bacterium to evolve solutions to CRISPR-Cas immunity. Furthermore, these Acrs are very similar (minimum 76.5% amino acid ID across all of them) to their homologs in virulent phages, in contrast to the *Listeria* AcrIIA4s (up to 41% ID to a temperate phage Acr).

There are currently four groups of genetically distinct *S. thermophilus* phages^30,33–35^, but there was noclear association between the presence or absence of a known Acr and a phage group (Fig. 2b)., Nor could we correlate the presence of an AcrIIA family in a phage to the CRISPR-Cas systems present in its hosts (Fig. 2c). Across 90 phages carrying *acrIIA6*, 48 different protein alleles were observed (Supplementary Fig. 1), while in 14 *acrIIA5*-carrying phages, we identified 11 unique protein alleles (Supplementary Fig. 2). No AcrIIA5 and AcrIIA6 shared any similarity. This discovery of two distinct families of Acrs disabling *S. thermophilus* CRISPR immunity is not unprecedented: AcrIIA1-4 are all very different but impede the same *L. monocytogenes* Cas9^24^.

### Structure of AcrIIA6

**Fig. 3:**
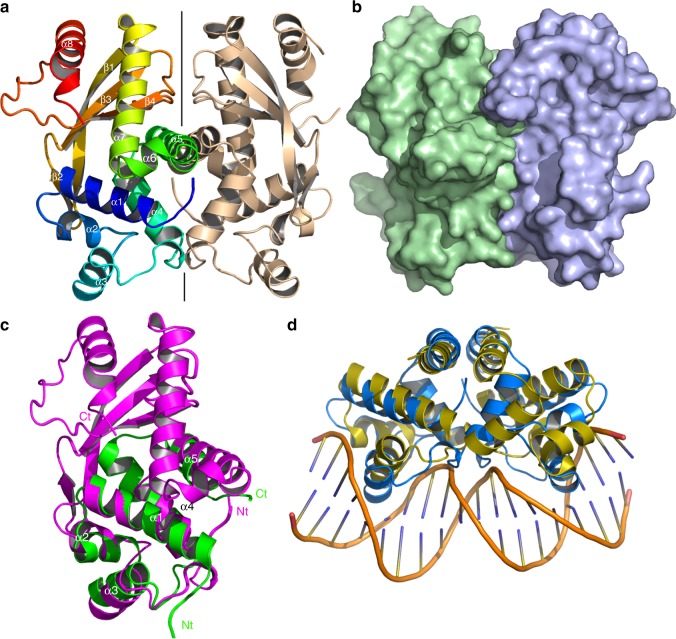
Crystal structure of AcrIIA6. **a** Ribbon view of the AcrIIA6 dimer. The left monomer is rainbow-colored, the right monomer is colored in beige. The secondary structures are identified α1–α8, β1–β4. **b** Surface representation with the same orientation and colors as in **a**. **c** Superimposition of AcrIIa6 monomer A (magenta) on a monomer of a putative transcription factor (PDB 2ef8, green). Helices α1–α5 are structurally aligned. **d** Superimposition of the AcrIIa6 dimer (blue) on the controller protein C.Esp1396I operator (yellow) in complex with dsDNA (orange; PDB 3s8q^37^). Both helices α3 are inserted in adjacent major grooves

With bioinformatic predictions yielding little information (Fig. 2a, c), we set about the biochemical characterization of AcrIIA6. It crystallized readily and its structure was solved at 1.96 Å revealing a dimeric assembly (Fig. 3, Supplementary Table 1). Gel filtration profiles also indicated that AcrIIA6 is a dimer in solution (Supplementary Fig. 3). Each monomer consists of eight α-helices and four β-strands forming a β-sheet, with the sequence topology α1α2α3α4α5α6α7β1β2β3β4α8 (Fig. 3a, b). Two other Acrs have been reported to form dimers^19,20^: AcrF3 interacts with the Cas3 subunit of type I-F CRISPR-Cas systems, which is not present in type II-A systems, while AcrIIA1 was found to co-purify with RNA, leading the authors to suggest that it interacts with nucleotides directly.

When the AcrIIA6 structure was submitted to the DALI server^36^, only low scores of structural similarity were returned. The best score (*Z* = 4.1 and rmsd of 2.3 Å for 71 aligned residues) was obtained with the HTH domain of an *Escherichia coli* putative transcription factor (PDB 2ef8). Superimposition of helices 1–5 from the AcrIIA6 monomer onto the 2ef8 monomer confirms an HTH domain in the Acr (Fig. 3c). This feature was not detectable by bioinformatic analyses of the protein sequence (Fig. 2a). The AcrIIA6 HTH domain is part of the dimerization interface, which is also similar to that of the bona fide HTH domain present in an *E. coli* controller protein C.Esp1396I solved in complex with dsDNA (PDB 3s8q)^37^. The superimposition of the AcrIIA6 HTH dimer onto the C.Esp1396I-dsDNA complex brings the AcrIIA6 helix 3 into contact with the two major grooves of the dsDNA (Fig. 3d). Helix 3 exhibits strong electropositivity, a feature observed in dsDNA binding helices, including the one from C.Esp1396I. Bio-layer interferometry (BLI) indicated binding to dsDNA, ssDNA, and a dsDNA-ssRNA duplex (Supplementary Fig. 4), but we do not know at this time if that affinity is altered by interaction with St1Cas9. A very recent study of the dimerizing AcrIIA1 revealed that it also contains an HTH domain^20^, and co-purifies with RNA when over-expressed in *E. coli*. Aside from this similarity the two structures differ considerably, as AcrIIA1 is formed exclusively of α-helices. We could not dock the AcrIIA6 structure to that of St1Cas9, as the latter is not available. We mapped the sequence conservation among the 48 unique AcrIIA6 alleles to the structure obtained (Supplementary Fig. 1). The dimerization interface is largely conserved as are the DNA-interacting arms of the HTH motif.

### Comparing AcrIIA activities

Since AcrIIA5 and now AcrIIA6 were discovered in phages infecting the *S. thermophilus* strain DGCC7854, a host containing only a single type II-A system (CR1, associated with St1Cas9), we decided to investigate the activities of Acrs orthologs from virulent phages infecting *S. thermophilus* hosts with two distinct type II-A systems; CR1 and CR3 (associated with St3Cas9). We reasoned these orthologs might have broader specificity. For AcrIIA5, we chose an ortholog present in phage D1126 (87% aa ID), whose host, DGCC7710, is a model for CRISPR immunity and whose CR1 and CR3 systems are well characterized, with the former being more active^38^. For AcrIIA6, we chose an ortholog (92% ID) from the reference phage DT1^39^ whose host, SMQ-301, also contains both CR1 and CR3 systems. We were also surprised to find an AcrIIA6 ortholog (92% ID) in phage D1024, which had displayed limited Acr activity in our earlier assays (Fig. 1a)—suggesting that the few differences between these two Acrs might be mechanistically relevant. The sequences of these 5 Acrs are compared in Supplementary Fig. 5. Furthermore, we compared the activities of these Acrs to those of the *L. monocytogenes* Acrs, AcrIIA2, and AcrIIA4. We cloned each of them into a vector for expression in two lactic acid bacteria. We then challenged *S. thermophilus* cells, immunized through either CR1 (St1Cas9) or CR3 (St3Cas9) system, or *Lactococcus lactis* cells immunized using an exogenous plasmid-borne SpCas9, with their respective virulent phages 2972 (*S. thermophilus*) or p2 (*L. lactis*). Of note, SpCas9 (1368 aa) is more similar to St3Cas9 (1409 aa, 57% ID) than to the shorter St1Cas9 (1121 aa, 17% ID).

**Fig. 4:**
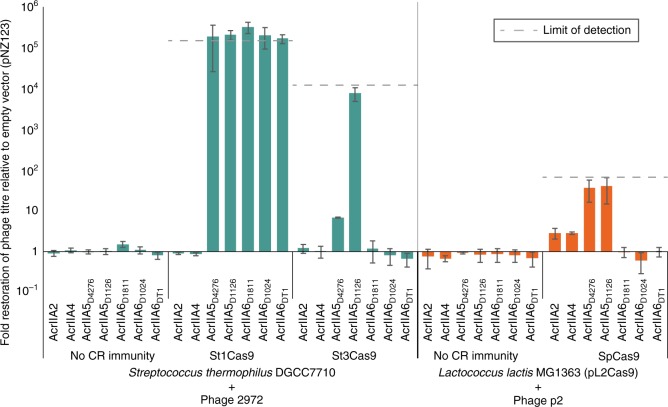
Comparison of type II-A Acr activities. Fold restoration of phage titres on strains carrying an Acr over ones carrying only the empty vector. The size of the bar reflects how effective the Acr is at bypassing the CRISPR immunity present, and each is the average of three biological replicates, each of three technical triplicates. The error bars represent the standard deviation. The leftmost three sections (green) are the relative titres of phage 2972 when its host is not immunized, or immunized at the endogenous CR1 or CR3 locus. The rightmost two sections (orange) are the relative titres of phage p2 when its host is not immunized, or immunized using an exogenous plasmid-borne SpCas9. Each system (CR1, CR3, SpCas9) varies in effectiveness, so the threshold limits of detection for each is indicated by a dotted line

All AcrIIA6 and AcrIIA5 orthologs completely restored the plaquing efficiency of phages targeted by the CR1 system of *S. thermophilus* (Fig. 4), indicating they are blocking the activity of St1Cas9. It is noteworthy that *S. thermophilus* phage D1024, which is carrying a distinct allele (*acrIIA6*_*D1024*_*)* in its genome, is only partially capable of bypassing CR1 immunity natively (Fig. 1a)—the increased effectiveness observed with the plasmid-borne *acrIIA6*_*D1024*_ version is likely reflective of a higher copy gene number, and its expression prior to phage entry. Similarly, the CRISPR-resistant phage D1811 (Fig. 1c) titres increased when its Acr was expressed from a plasmid. Neither *Listeria* AcrIIA2 nor AcrIIA4 displayed any effectiveness against the streptococcal CR1 system.

CR3-immunity was unaffected by any AcrIIA6 ortholog (Fig. 4), while both AcrIIA5s were effective against the CR3 system (Fig. 4). Notably, AcrIIA5_D4276_ comes from a phage infecting a *S. thermophilus* host which lacks the CR3 locus, and its activity is limited to a 10-fold inhibition of CR3 activity^25^. In contrast, AcrIIA5_D1126_ (87% ID with AcrIIA5_D4276_), which originates from a phage infecting a strain (DGCC7710) carrying both CR1 and CR3 systems, can completely inhibit St3Cas9’s activity—the first reported Acr to do so.

We also tested the seven AcrIIAs for their ability to impede an exogenous plasmid-borne SpCas9 in the *L. lactis* model. AcrIIA6s did not impede SpCas9, while AcrIIA2 and AcrIIA4 exhibited only a partial inhibition. Both AcrIIA5s almost completely abolished SpCas9 activity in this bacterial system (Fig. 4). We had previously reported that this lactococcal phage assay is leaky^25^, with plaque morphology also corresponding to the effectiveness of the Acr. Plaque sizes corresponded with the efficacy observed in Fig. 4 (Supplementary Fig. 6).

### Genome-editing applications

The lactococcal SpCas9 assay, though very useful for side-by-side comparisons of Acrs, has shortcomings for evaluating genome-editing potential. In addition to the non-eukaryotic background, the assays are performed at 33 °C, close to *L. lactis*’ optimal growth temperature (30 °C), rather than at 37 °C. Therefore, we evaluated the effectiveness of the seven AcrIIAs against the two more distant relatives, SpCas9 and St1Cas9, in human K562 cells. In addition to SpCas9, St1Cas9 has been used previously in genome-editing applications, due to its different PAM specificity^40^. Following transient overexpression of the Cas9s and Acrs, we determined the frequency of Cas9-induced indels in treated cells using the Surveyor nuclease^41^.

**Fig. 5:**
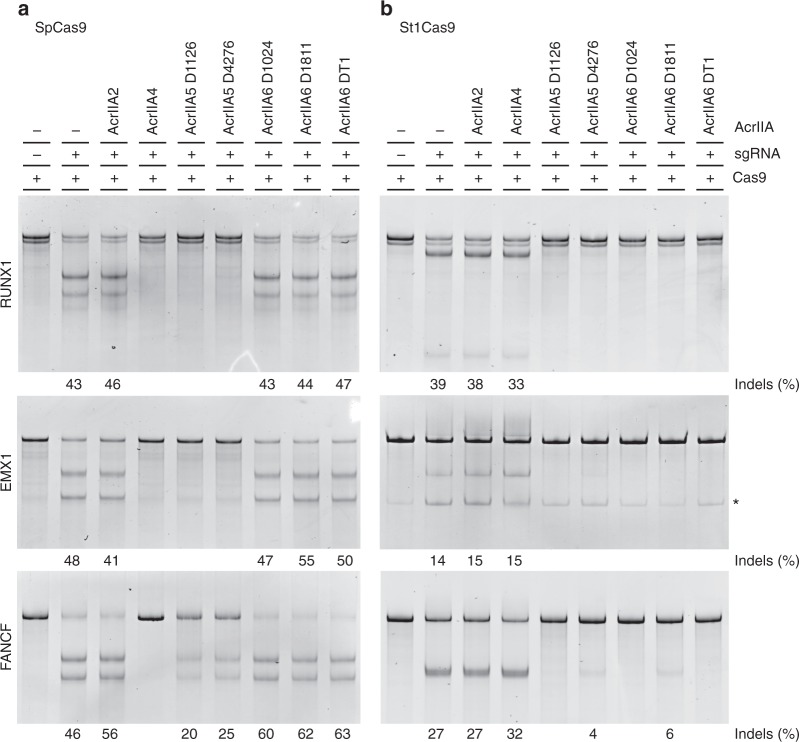
Anti-CRISPR activity of AcrIIAs against SpCas9 and St1Cas9 in human cells. **a** K562 cells were transfected with a dual SpCas9 and sgRNA expression plasmid (500 ng) targeting *RUNX1*, *EMX1,* or *FANCF*. Where indicated, a second vector (500 ng) was used to co-express human codon-optimized AcrIIAs. The Surveyor assay was performed 3 days later to determine the frequency of indels, as indicated at the base of each lane. **b** Same as in **a** but cells were transfected with an St1Cas9 expression vector (500 ng) in addition to an sgRNA expression plasmid (800 ng). (−) Indicates that the transfection mix contained the same amount of a related empty vector. * Indicates a non-specific band co-migrating with the specific cleavage product

In agreement with previous studies^8^, we observed near complete inhibition of SpCas9 activity at three different targets with AcrIIA4 (Fig. 5a). However, AcrIIA4 demonstrated no activity against St1Cas9 (Fig. 5b). This inhibition profile is consistent with its known mode of action: interacting with the PAM-recognition domain of SpCas9^27,28^. As SpCas9 and the Cas9 from *Listeria* require the same (NGG) PAM, share the same DRKRYT specificity motif, and are identical at 15 (similar at 17) of the 19 residues identified as interacting with AcrIIA4^27,28^, AcrIIA4 robustly inhibits SpCas9 but has no effect on Cas9 proteins recognizing other PAMs.

None of the three AcrIIA6 orthologs had any detectable effect on SpCas9 (Fig. 5a), confirming the results observed with the *L. lactis* system, but all three were capable of inhibiting St1Cas9, with residual nuclease activity observed at only one target for AcrIIA6_D1811_. Further validating the *L. lactis* system, we observed no detectable activity of AcrIIA2 against any Cas9 in this assay. While AcrIIA2 and SpCas9 do interact in vitro^27,30,42^, our data show that these interactions are not always indicative of utility in the context of genome-editing applications. Some effectiveness of AcrIIA2 on SpCas9 was previously demonstrated in HEK293T cells^24^. It should be noted that the levels of inhibition observed might not only reflect the potency of the Acrs, but also their expression and functionality in these ectopic contexts. Notably, the steady state levels of the AcrIIAs expressed from the *AAVS1* safe harbor locus in human cells varied (Supplementary Fig. 7).

Of all the Acrs tested in human cells and in the bacterial assays, AcrIIA5 displayed the broadest range of activity (Figs. 4, 5). It could completely inhibit SpCas9 at two targets, providing ~60% inhibition at the other site, and also proved to be an equally potent inhibitor of St1Cas9. The activities of all tested Acrs in our assays are summarized in Table 1. A concurrent in vitro study also observed a wide range of AcrIIA5 activities, stretching so far as to inhibit the type II-C Cas9 proteins of *Neisseria meningitidis* and *Campylobacter jejuni*^42^.

**Table 1:** Summary of AcrIIA activities

		Bacterial Assays			Human Assays	
		*S. thermophilus* DGCC7710	*S. thermophilus* DGCC7710	*Lactococcus lactis* MG1363	K562 cells	K562 cells
		St1Cas9 (1121 aa)	St3Cas9 (1409 aa)	SpCas9 (1368 aa)	SpCas9 (1368 aa)	St1Cas9 (1121 aa)
AcrIIA2 (123 aa)	*L. monocytogenes* prophage	**−**	**−**	**+**	**−**	**−**
AcrIIA4 (87 aa)	*L. monocytogenes* prophage	**−**	**−**	**+**	**+++**	**−**
AcrIIA5 D1126 (140 aa)	*S. thermophilus* phage D1126	**+++**	**+++**	**+++**	**++**	**+++**
AcrIIA5 D4276 (140 aa)	*S. thermophilus* phage D4276	**+++**	**+**	**+++**	**++**	**++**
AcrIIA6 D1024 (183 aa)	*S. thermophilus* phage D1024	**+++**	**−**	**−**	**−**	**+++**
AcrIIA6 D1811 (183 aa)	*S. thermophilus* phage D1811	**+++**	**−**	**−**	**−**	**++**
AcrIIA6 DT1 (183 aa)	*S. thermophilus* phage DT1	**+++**	**−**	**−**	**−**	**+++**

## Conclusions

Delving into the phages of *S. thermophilus* has yielded a new Acr family, AcrIIA6, and an associated structure. While 38% of the virulent *S. thermophilus* phages analyzed contain AcrIIA5 or AcrIIA6, surprisingly all temperate *S. thermophilus* phages known to date possess one of these two AcrIIAs. These findings confirm that virulent phages can carry Acrs, and, in contrast with previous findings, the Acrs are closely related between virulent and temperate phages. This may be a result of stronger selection for Acr-containing virulent phages from repeated use of CRISPR-immunized strains in industrial settings. Acrs with orthologs in virulent phages have also consistently proven effective in genome-editing applications. AcrIIA5 displays the broadest type II-A Acr activity to date, in bacteria, in human cells, and in vitro. Our findings support the notion that the phage-host interactions in *S. thermophilus*, and possibly other bacterial systems, are strongly influenced by Acrs. For example, the CR3 system of *S. thermophilus* strain SMQ-301 was thought to be inherently more active than the co-occurring CR1 system, but this is a result of the AcrIIA6 carried by the phage used to challenge it, DT1. This raises interesting questions: *S. thermophilus* strains typically contain up to four CRISPR-Cas systems—two have been considered dormant because phage challenges have never revealed acquisition of new spacers at these loci. It is tempting to speculate that these loci appear silent because Acrs are far more widely spread than initially suspected in *S. thermophilus*.

## Methods

### Bacterial cultures

Strains and plasmids used in this study are listed in Supplementary Table 2. *S. thermophilus* cultures were grown in M17 medium (Oxoid) with 0.5% w/v lactose (LM17). If the plasmid pNZ123 was present, 5 μg ml^−1^ chloramphenicol was added to the medium. Overnight *S. thermophilus* cultures were grown at 37 °C without shaking, but same-day cultures were grown at 42 °C. *L. lactis* cultures were grown in M17 medium with 0.5% w/v glucose (GM17). If the plasmid pNZ123 or pL2Cas9 was present, 5 μg ml^−1^ chloramphenicol or erythromycin was added, respectively. *L. lactis* cultures were grown at 30 °C without shaking. If phages were to be added, the medium was further supplemented with 10 mM CaCl_2_. *E. coli* cultures were grown in LB medium at 37 °C with shaking. If the pNZ123 plasmid was present in the *E. coli* strain, 20 μg ml^−1^ chloramphenicol was added.

### Phage amplification and titration

Scrapings of phage lysates preserved at −80 °C with 15% glycerol were co-inoculated with their host strain until complete lysis occurred. This lysate was then filtered through a 0.45 μm PES filter and 100 μl of the filtrate was used to inoculate the host strain grown to an OD_600_ of 0.1. This second amplification lysate was also filtered (0.45 μm PES filter) and stored at 4 °C. Phage titres were obtained through serial dilutions of phage lysate in buffer (50 mM Tris-HCl, pH 7.5, 100 mM NaCl, 8 mM MgSO_4_). One hundred microliters of diluted phage lysate and 300 μl of an indicator strain grown to an OD_600_ of 0.6 were co-inoculated into 3 ml of molten 0.75% agar medium at 55 °C. The molten mix was then rapidly poured onto a plate of the same medium with 1% agar, and allowed to set. The plates were incubated overnight, and plaques were counted on those plates with dilutions resulting in between 30 and 300 plaques. For small lactococcal plaque counting as shown in Supplementary Fig. 6, 0.4% molten LE agarose was used instead, and incubation was done at 33 °C to reach a temperature closer to *S. pyogenes* optimal growth temperature (source of SpCas9).

### Immunizing assays

Phages were diluted in phage buffer in order to obtain a final multiplicity of infection of 0.1 plaque-forming unit per colony forming unit (pfu/cfu). Phages were plated with *S. thermophilus* DGCC7854 as described above for phage titres, but surviving colonies (rather than plaques) were counted. Surviving colonies (bacteriophage insensitive mutants) were screened by PCR for acquisition of new spacers at the CR1 locus (DGCC7854) using primers described in Suplementary Table 2. An increase in the size of the PCR product relative to the wild type was indicative of CRISPR immunization.

### Phage genome sequencing and annotation

The DNA of phage D1811, D1024, and D5842 was extracted using a PureLink Viral RNA/DNA kit (Invitrogen, MA, USA), and sequenced on a MiSeq system using a MiSeq reagent kit v2 after preparation using the Nextera XT DNA library preparation kit (Illumina, British Columbia, Canada). The genome was assembled using Ray v. 2.2.0^43^ and annotated through GeneMark.hmm prokaryotic^44^ and ORF-FINDER^45^. These annotations were manually curated based on comparisons to related phages. The annotated genomes were deposited in GenBank (accession numbers listed in Supplementary Table 2).

### Phage gene cloning for each pNZAcr construct

Primers (listed in Supplementary Table 2) were designed to amplify the *S. thermophilus* phage *acr* genes of interest and their ribosome binding site, as well as to add 30-nt extensions overlapping the pNZ123 multiple cloning site to facilitate Gibson assembly. In the case of the AcrIIA2 and AcrIIA4 synthesized constructs, the upstream region was replaced with that of AcrIIA5 to ensure a ribosome binding site known to be functional in lactic acid bacteria, and a single nucleotide substitution (C-A) was made to avoid the rare CGC arginine codon. The amplified gene was then cloned by Gibson reaction into pNZ123 digested with XbaI. The resulting plasmid was transformed into commercial NEB5a *E. coli* cells according to the manufacturer’s recommendations (New England Biolabs, Ontario, Canada), then purified using a Qiaprep Spin Miniprep kit. The purified plasmid was then transformed into the relevant *S. thermophilus* and *L. lactis* strains through a glycine shock-based protocol followed by electroporation^29,46^. The sequence of the insert was confirmed by sequencing using primers pNZins_F and pNZins_R.

### AcrIIA5 and AcrIIA6 analyses

The protein sequences of AcrIIA5 and AcrIIA6 were subjected to analysis through BlastP^47,48^, HTH prediction^49^, pcoils^50^, jPred4^51^, and hhpred^50^. Their orthologs in phage genomes were identified using BlastN^48^.

### Protein expression and purification

AcrIIA6 was subcloned using the Gateway cloning system into a bacterial expression vector pETG-20A (with an *N*-terminal 6His-TRX-tev tag). The protein was overexpressed in *E. coli* Rosetta pLyS cells cultured in NZY auto-induction medium (NZYTech) at 25 °C for 24 h. Cells were harvested by centrifugation (4000 × *g* for 10 min), and the pellet was homogenized and frozen in lysis buffer (20 mM Hepes pH 7.5, 150 mM NaCl, 5 mM imidazole, 0.1 mg ml^−1^ lysozyme, 1 mM phenylmethylsulfonyl fluoride (PMSF)). After thawing, DNAse I (20 μg ml^−1^) and MgSO_4_ (1 mM) were added and cells were lysed by sonication. The pellet and soluble fractions were separated by centrifugation (16,000 × *g* for 30 min). The soluble fraction from the lysate was purified by affinity chromatography using a 5 ml HisTrap FF crude (GE Healthcare) Ni^2+^-chelating column equilibrated in buffer A (20 mM Tris pH 8.0, 150 mM NaCl, 5 mM imidazole). The protein was eluted with buffer A supplemented with 250 mM imidazole and was further purified by a size exclusion chromatography (HiLoad 16/600 Superdex 200 prep grade, GE) equilibrated in 20 mM Hepes pH 7.5, 150 mM NaCl. The 6His-TRX tag was removed by addition of 1:10 (mol:mol) of TEV protease and incubation for 48 h at 20 °C. The cleaved AcrIIA6 protein was eluted from HisTrap FF crude Ni^2+^-chelating column and was further separated by size exclusion chromatography (Hiload 26/600 Superdex 75 prep grade, GE Healthcare) with 20 mM Hepes pH 7.5, 150 mM NaCl. Purity of the protein was monitored at all stages of the purification using SDS–PAGE, and visualized by Coomassie blue staining.

### Crystallization and structure determination

The purified AcrIIA6 was concentrated to 7 mg ml^−1^ using an Amicon concentrator (Millipore). Initial crystallization trials of AcrIIA6 were performed by the sitting-drop vapor-diffusion method at 293 K in 96-well Swissci plates using a Mosquito Crystal robot (TTP Labtech) with the following screens: Stura Footprint Screens (Molecular Dimensions), Pact1er (Molecular Dimensions), Wizard I and II (emerald), JCSG + Suite (Qiagen/Nextal), and amSO4 Suite (Qiagen/Nextal). Crystallization hit occurred in condition No. B8 of the JCSG + Suite [200 mM Magnesium chloride, 100 mM Tris pH 7, 10% (w/v) PEG 8000]. After optimization^52^, the final crystallization conditions were 0.2 M magnesium chloride, 0.1 M Tris pH 6.5–7.5, 5–15% (w/v) PEG 8000. Crystals were briefly soaked in crystallization solution supplemented with 20% (v/v) propylene glycol. Before flash cooling in liquid nitrogen, a heavy atom derivative was produced by soaking crystals in a solution of tantalum bromide cluster (Ta_6_Br_12_:Br_2_), leading to crystals becoming green in few seconds. A fluorescence scan was performed to determine the peak wavelength (0.9882 Å). Single-wavelength anomalous diffraction data were collected to 2.9 Å resolution on Ta_6_Br_12_:Br_2_ soaked AcrIIA6 crystals at beamline Proxima-1 at SOLEIL (Paris, France). Native AcrIIA6 diffraction data sets were collected on beamline Proxima-1 at Soleil, Paris, France (Supplementary Table 1). The data sets were integrated with XDS^53^ and were scaled with SCALA from the CCP4 Suite^54^. Then, auto-tracing was performed with BUCCANEER^55^ and several iterations of model improvement were conducted by cycling through refinement with autoBUSTER^56^, phase improvement by density modification with PARROT^54^, auto-tracing with BUCCANEER^55^ and manual pruning and rebuilding using COOT^57^. The cubic and tetragonal structures have 98.1% and 97.2% aa in the preferred regions of the Ramachandran plot, and no residue in the outliers regions. The refinement statistics are presented in Supplementary Table 1. A stereo image of a portion of the electron density map is available in Supplementary Figure 8.

### Bio-layer interferometry (BLI)

AcrIIA6 binding to dsDNA, DNA-RNA heteroduplex, and ssDNA was measured using BLI. A 20-mer biotinylated target ssDNA and its DNA and RNA complements were purchased from Integrated DNA Technologies (IDT) (Bio-ssDNA: 5′-AATACTTTTATCAACGCAAG-3’; ssDNA: 5′-CTTGCGTTGATAAAAGTATT-3′; ssRNA: 5′-CUUGCGUUGAUAAAAGUATT-3′). Oligo strands were resuspended in duplex buffer (30 mM HEPES pH 7.5, 100 mM potassium Acetate), mixed in equal molar amounts, heated to 94 °C for 2 min, and gradually cooled down to form double-stranded targets according to IDT protocol. Streptavidin-coated biosensors were used to immobilize biotinylated ligands and monitor association and dissociation of AcrIIA6. All experiments were performed using an Octet® Red96 system at 27 °C, with an agitation speed of 1000 rpm in the interaction buffer (50 mM HEPES pH 7.5, 100 mM KCl, 2 mM MgCl2, 0.01% Tween 20, 0.01 mg m^−1^ BSA). The consecutive steps of the BLI measurements were as follows: 60 s incubation in buffer, 180 s in 250 nM of duplexes (ligand loading to a signal of 0.7 nm), 60 s incubation in biocytin (quenching of biosensors surface), 60 s wash in buffer, 120 s baseline measurement, 340 s association in 6 μM AcrIIA6, and 300 s dissociation in buffer. Association and dissociation curves were double referenced against the buffer reference signal (biosensors coated with ligands and incubated in buffer only), and the reference sensors signal (biosensors without ligands and incubated in AcrIIA6). Data were analysed using the Fortebio Data Analysis software 8.2.

### Cell culture and transfection

K562 cells were obtained from the ATCC and maintained at 37 °C under 5% CO_2_ in RPMI medium supplemented with 10% FBS, penicillin-streptomycin, and GlutaMAX. Cells (2E5 per transfection) were transfected using the Amaxa 4D-Nucleofector (Lonza) per manufacturer’s recommendations. Targeted integration to the *AAVS1* locus was achieved by co-transfection of ZFNs (500 ng) and donor plasmids (1 µg)^58^. Pools of cells were selected for 10 days with 0.5 µg ml^−1^ puromycin starting 3 days post-transfection.

### Plasmids and oligonucleotides used for K562 cell work

SpCas9 and associated sgRNAs were expressed from pX458^5^ (Addgene plasmid #48138, a gift from Feng Zhang). Human expression vectors for *S. thermophilus* St1Cas9 and associated sgRNA were a gift from Keith Joung (Addgene plasmids #65775 and #65778)^59^. Spacer (guide) sequences for SpCas9 and St1Cas9 have been described previously^59,60^ and are provided in Supplementary Table 3. Untagged AcrIIA proteins were expressed transiently from a modified pVAX1 vector (Thermo Fisher Scientific) containing a beta-globin intron. The AcrIIA ORFs were codon-optimized for expression in human cells and synthesized as gBlocks gene fragments (IDT) (Supplementary Table 4). To establish cell lines stably expressing the various AcrIIAs, gBlock gene fragments were cloned into AAVS1_Puro_PGK1_3xFLAG_Twin_Strep (Addgene plasmid #68375)^58^.

### Surveyor nuclease assays

Genomic DNA was extracted from 2.5E5 cells with 250 µl of QuickExtract DNA extraction solution (Epicentre) per manufacturer’s recommendations. The various loci were amplified by PCR using the primers described in Supplementary Table 5. Assays were performed with the Surveyor mutation detection kit (Transgenomics) as described^41^. Samples were separated on 10% PAGE gels in TBE buffer. Gels were imaged using a ChemiDoc MP (Bio-Rad) system, and quantifications were performed using the Image lab software (Bio-Rad).

### Flow cytometry

The frequency of mScarlet-I expressing cells was assessed with a BD LSR II flow cytometer.

### Data availability

Coordinates and structure factors for AcrIIA6 in a cubic native and tetragonal native form have been deposited in the RCSB Protein Data Bank under accession codes 6EYY and 6EYX, respectively. Nucleotide sequences for genomes of phages D5842, D1024, and D1811 have been deposited in GenBank under accession codes MH000602, MH000603, and MH000604, respectively. Other relevant data supporting the findings of the study are available in this article and its Supplementary Information files, or from the corresponding author upon request.

## Electronic supplementary material

Supplementary Information
