# Supplementary material for: Widespread anti-CRISPR proteins in virulent bacteriophages inhibit a range of Cas9 proteins
Source: Nat Commun. 2018 Jul 25;9:2919. doi: 10.1038/s41467-018-05092-w (PMC6060171; doi:10.1038/s41467-018-05092-w)
Supplement: Supplementary file 1 — Supplementary Information [file 41467_2018_5092_MOESM1_ESM.pdf]

Widespread anti-CRISPR proteins in virulent bacteriophages inhibit a range of Cas9 proteins

Hynes et al.

Supplementary Information

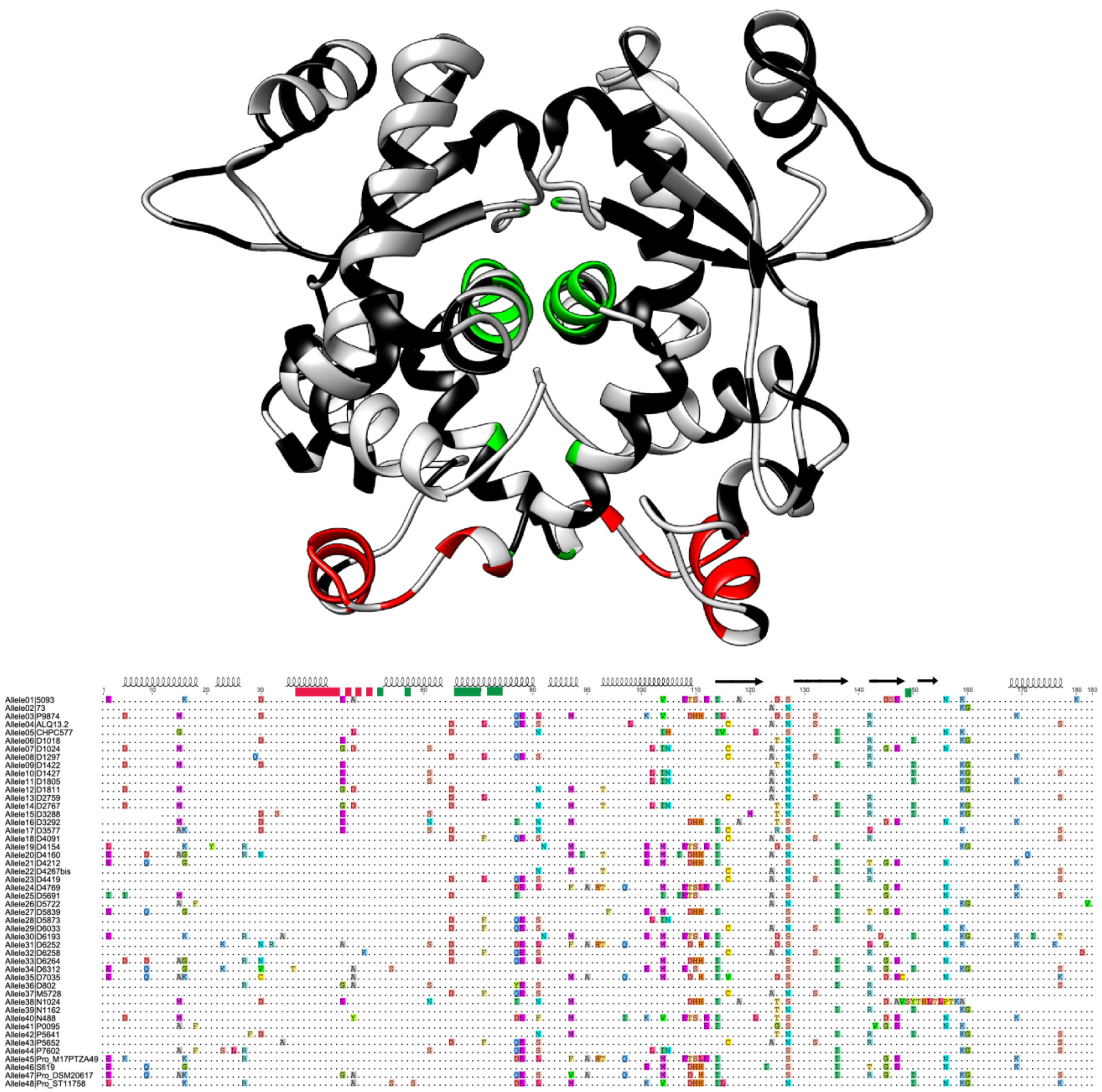

Supplementary Fig. 1. Sequence conservation for 48 different alleles of AcrIIA6. (Top) The sequence conservation in 48 alleles of AcrIIA6 mapped onto the structure of the dimer. Each Allele is identified with a number and the name of the phage it was found following the vertical bar. Residues are white if not 100% conserved, and black, red (HTH DNA-binding interface) or green (dimerization interface) if conserved. (Bottom) Alignment of all 48 alleles of AcrIIA6 using Geneious v7.1[1], with residues coloured by amino acid similarity. A dot (.) indicates identity to the consensus sequence (not depicted). Loops indicate α-helical residues, black arrows indicate β-strands, as represented by ESPript[2]. The red and green boxes above the alignment correspond to the colouring visible on the structure (Top).

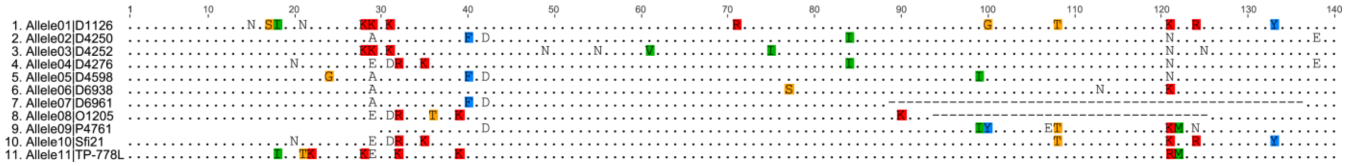

Supplementary Fig. 2. Sequence conservation for 11 different alleles of AcrIIA5. Alignment of all 11 protein alleles of AcrIIA5 using Geneious v7.1[1]. Each Allele is identified with a number and the name of the phage it was found following the vertical bar. Residues are coloured by amino acid similarity. A dot (.) indicates identity to the consensus sequence (not depicted), while a dash (–) indicates a residue not present in the sequence, but present in the consensus.

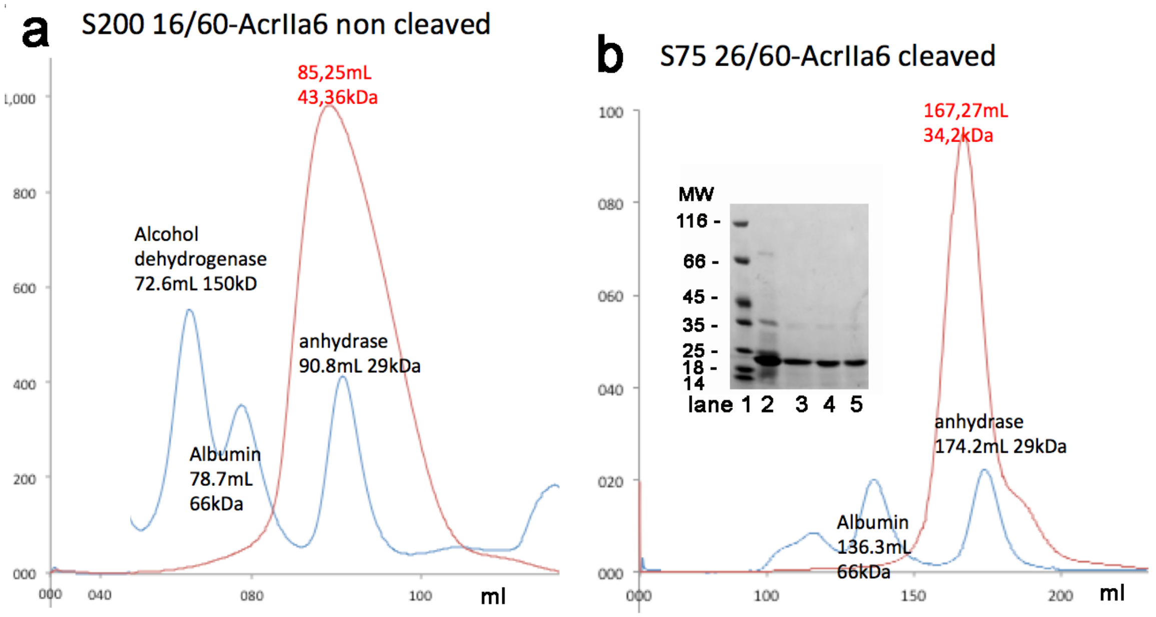

Supplementary Fig. 3. Size exclusion chromatography of the His$_6$-Trx-TEV-AcrIIA6 construct. (a) before cleavage (column S200 16/60) and (b) after cleavage (column S75 26/60). The fusion construct His$_6$-Trx-TEV-AcrIIA6 migrates as a monomer (theoretical MW: 36.1 kDa) while AcrIIA6 migrates as a dimer at ~40 kDa (theoretical MW of the monomer: 21.7 kDa), as judged from calibration curves. Inset: SDS gel of cleaved product. Lanes 1: molecular weight markers in kDa. Lane 2: output of the affinity column. Lanes 3-5: output of the SEC column. The AcrIIA6 monomer molecular weight is ~22 kDa.

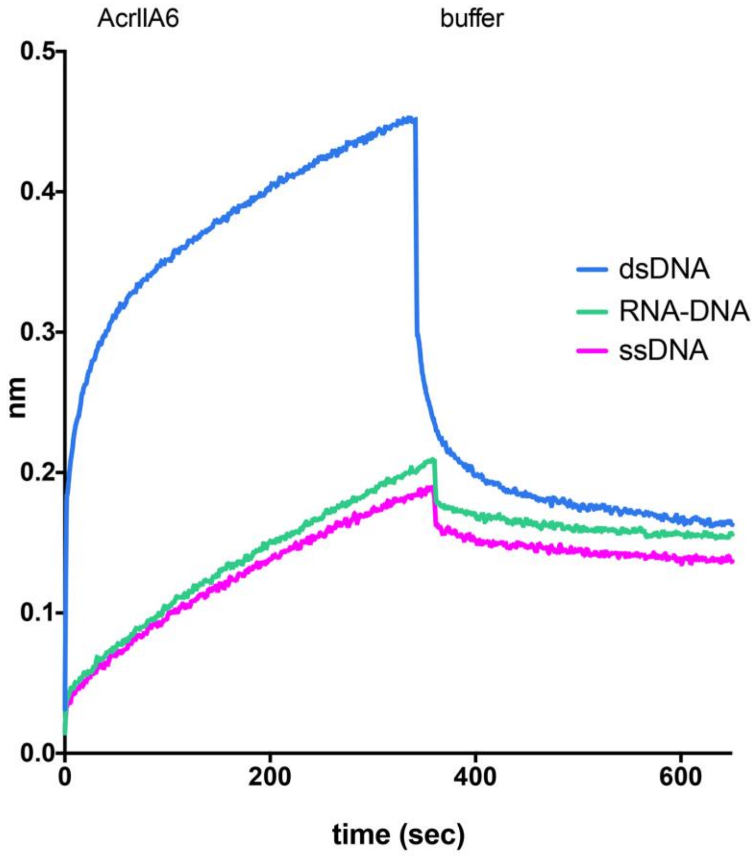

Supplementary Fig. 4. Binding assays between AcrIIA6 and oligonucleotides using Bio-Layer Interferometry. Biotinylated dsDNA, RNA-DNA heteroduplex and ssDNA of similar sequences were immobilized on streptavidin-coated biosensors and dipped into 6 μM of AcrIIA6. The AcrIIA6 and buffer labels indicate the association and dissociation steps, respectively.

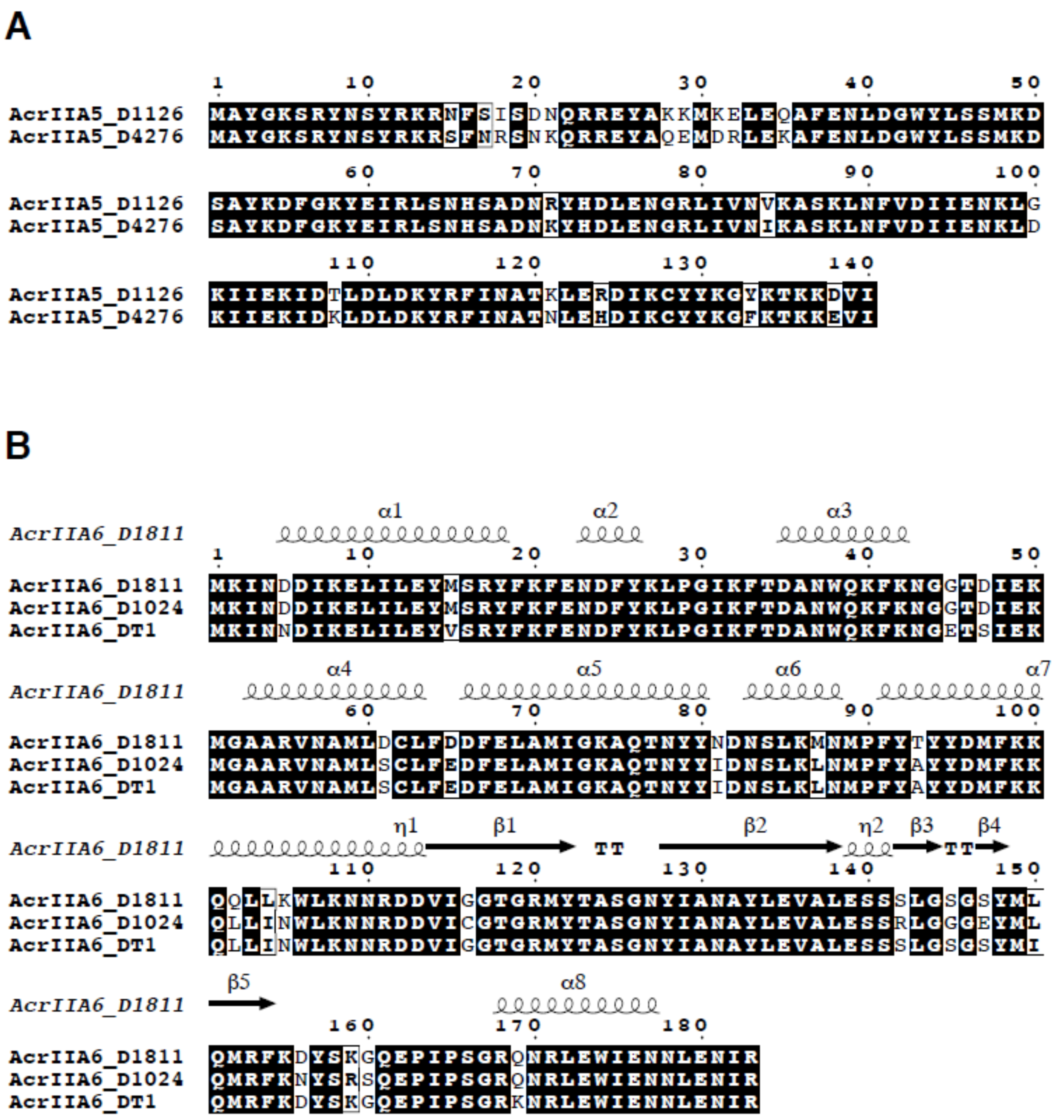

Supplementary Fig. 5. Amino acid alignment of the AcrIIAs selected for further investigation (in Figures 4 and 5). Residues numbered and highlighted according to identity (black), similarity (bolded & black outline), or difference (white) for AcrIIA5 (a) and AcrIIA6 (b). (b) The secondary structure of the associated residues in AcrIIA6$_{D1811}$ are depicted above (large loop= α-helix, small loop "η" = 3_{10}-helix, arrow = β sheet, TT = strict β-turns. Note β3-β4 appear as a single β3 sheet in Figure 3a), visualized using ESPript[2].

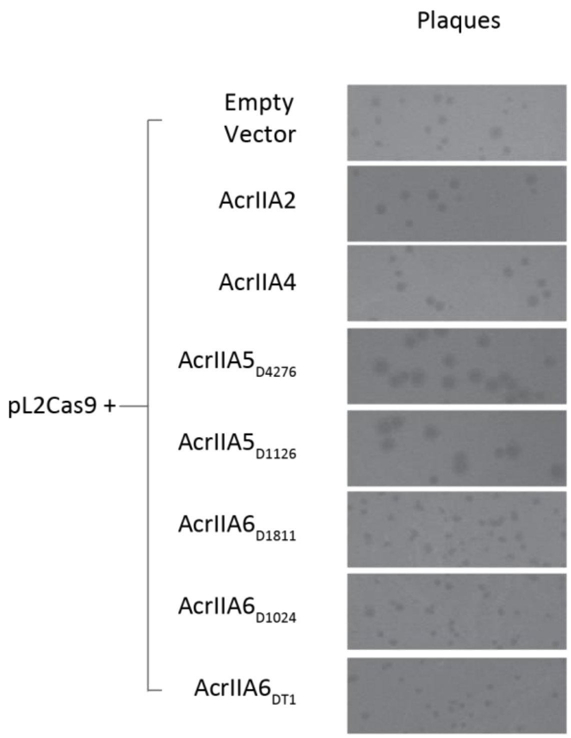

Supplementary Fig. 6. Plaque morphologies of p2 plaques leaking-through or bypassing immunity granted by pL2Cas9 in the presence of each tested AcrIIA. A representative section of a plate is presented (right) under identical magnification for each.

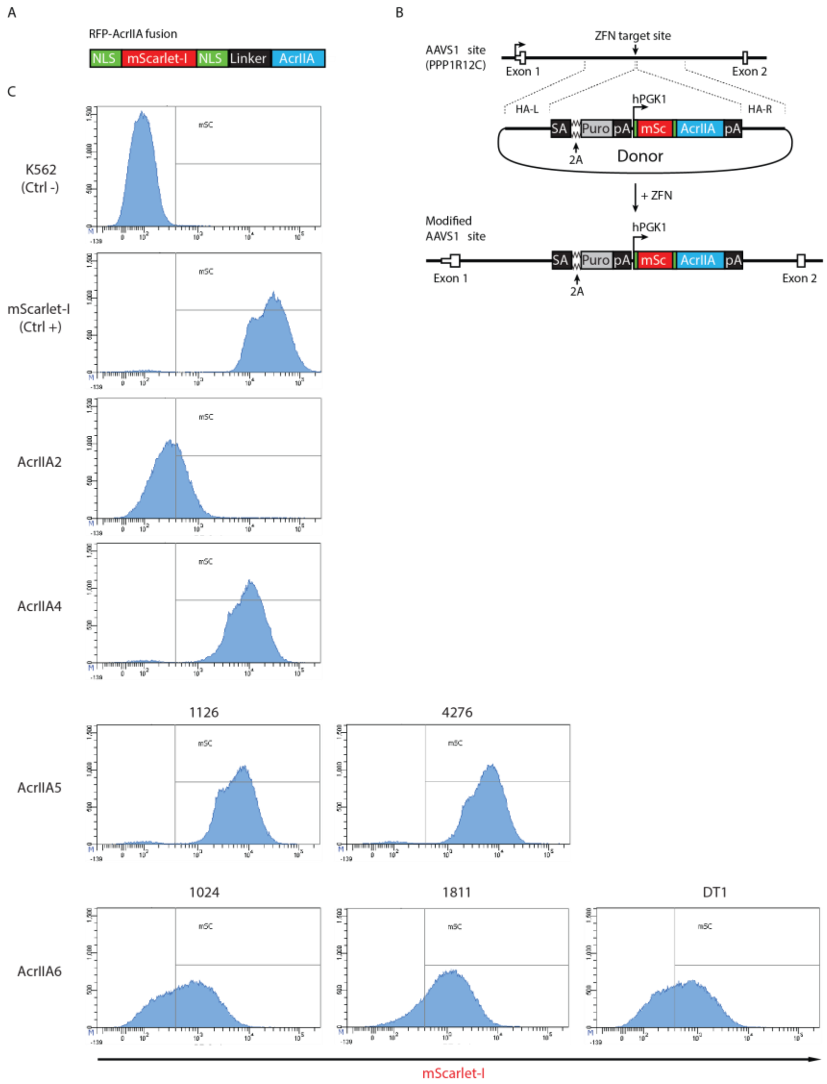

Supplementary Fig. 7. Steady state levels of AcrIIAs expressed constitutively from the *AAVS1* safe harbor locus. (a) Schematic of the AcrIIA protein fusions. AcrIIA ORFs were genetically fused at their N-terminus to monomeric Scarlet-I (mSc), a bright red fluorescent protein, and to nuclear localization sequences (NLS). (b) Schematic of the ZFN-driven targeted integration of mSc-AcrIIAs to the *AAVS1* locus. The first two exons of the *PPP1R12C* gene are shown as open boxes. Also annotated are the locations of the splice acceptor site (SA), 2A self-cleaving peptide sequence (2A), puromycin resistance gene (Puro), polyadenylation sequences (pA), human phosphoglycerate kinase 1 promoter (h*PGK1*), homology arms left and right (HA-L, HA-R) are respectively 800 and 840 bp. (**c**) FACS-based quantification of mSc-AcrIIA expression levels in cycling polyclonal populations of K562 cells obtained after puromycin selection.

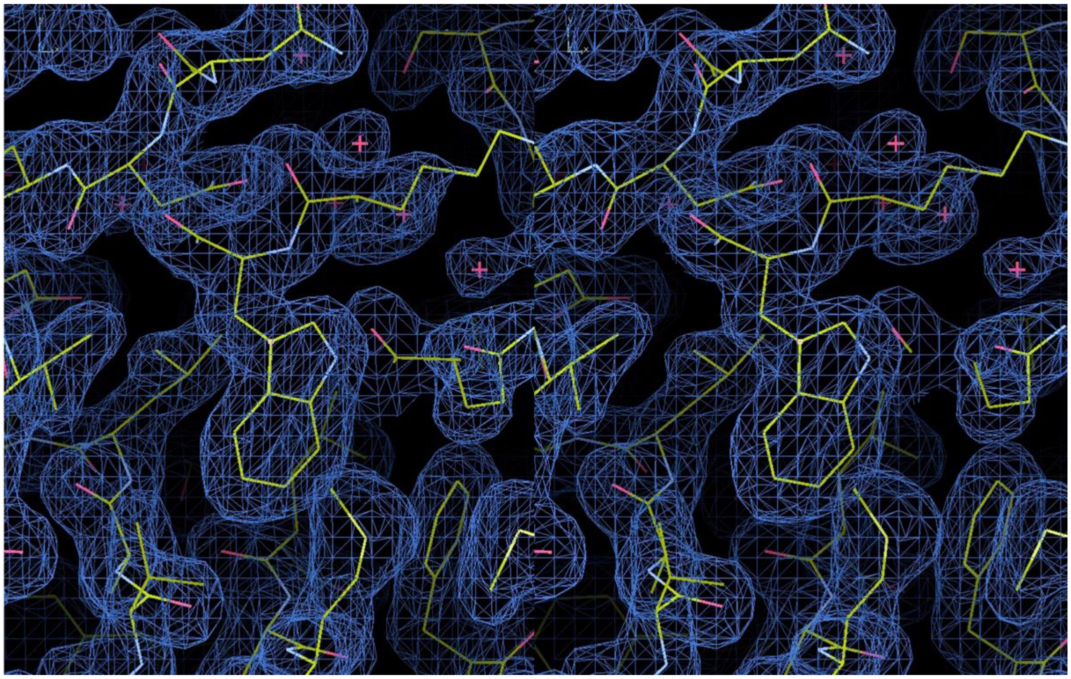

Supplementary Fig. 8. Stereo image of a portion of the electron density map (2Fo-Fc) contoured at 1 σ level around Trp 106 belonging to helix 7.

Supplementary Table 1. Data collection and refinement statistics of AcrIIA6 (values in parentheses are for highest-resolution shell).

	Ta_6Br_{12}#	Cubic native	Tetragonal native
**Data collection**			
Space group	$P4_332$	$P4_332$	$P4_32_12$
a, *b*, *c* (Å)	a=b=c=175.15	a=b=c=175.15	a=b=71.37, c=177.7
α, β, γ (°)	α=β=γ=90	α=β=γ=90	α=β=γ=90
Resolution (Å)	30-3.02 (3.23-3.02)*	50-2.5 (2.65-2.50)*	50-1.96 (2.1-1.96)*
R_{sym}	0.187 (1.54)	0.189 (1.0)	0.139 (1.17)
$I / \sigma I$	16.9 (2.5)	13.3 (2.2)	15.8 (3.54)
Completeness (%)	99.9 (99.9)	99.9 (99.9)	99.5 (99.2)
Redundancy	35 (35)	26 (26)	24 (24)
**Refinement**			
Resolution (Å)		48.8-2.55(2.58-2.50)	26.4-1.96 (1.96-2.02)
No. reflections		32398 (2946)	33785 (2875)
R_{work} / R_{free}		23.2/24.5(0.31/0.31)	0.213/0.227(0.216/0.246)
No. atoms			
Protein		3004	2950
Ligand/ion		0	0
Water		287	348
B-factors§			
Protein		65.0	38.6
Water		65.2	49.6
R.m.s. deviations			
Bond lengths (Å)		0.009	0.009
Bond angles (°)		1.08	1.01

* One crystal collected

# The working structure obtained from the dataset of the AcrIIa6 Ta_6Br_{12} derivative was only used for solving the higher resolution structures of the cubic and tetragonal forms by molecular replacement and was not refined nor deposited into the PDB.

§ Wilson *B*-factors are 78.1 and 37.5, respectively.

Supplementary Table 2. List of strains, plasmids and primers.

Strains	Description	Function	Source
NEB5α	*Escherichia coli* competent cells	Cloning	NEB
Rosetta pLyS	*Escherichia coli* competent cells	Cloning for overexpression	Novagen
DGCC7854	*Streptococcus thermophilus,* Host to 4 phages, CR1 adaptive	Amplification of 4 phages (Fig 1,2)	(3)
SMQ-1343	DGCC7854, CR1-programmed vs 4 phages	Finding an ACR-containing phage (Fig 1)	(3)
SMQ-1344	DGCC7854 (pNZ123) CmR	Control for ACR activity (Fig 1)	(3)
SMQ-1345	SMQ-1343 (pNZ123) CmR	Control for ACR activity (Fig 1)	(3)
SMQ-1359	NEB5a (pNZAcrIIA2), CmR	Source of plasmid pNZAcrIIA2	This study
SMQ-1360	NEB5a (pNZAcrIIA4), CmR	Source of plasmid pNZAcrIIA4	This study
SMQ-1361	NEB5a (pNZAcr-1811), CmR	Source of plasmid pNZAcr-1811	This study
SMQ-1362	NEB5a (pNZAcr-1024), CmR	Source of plasmid pNZAcr-1024	This study
SMQ-1363	NEB5a (pNZAcr-DT1), CmR	Source of plasmid pNZAcr-DT1	This study
SMQ-1364	NEB5a (pNZAcr-1126), CmR	Source of plasmid pNZAcr-1126	This study
SMQ-1365	DGCC7854 (pNZAcr-1811) CmR	Control for ACR activity (Fig 1)	This study
SMQ-1366	SMQ-1343 (pNZAcr-1811) CmR	Test for ACR activity (Fig 1)	This study
DGCC7710	*S. thermophilus,* host to 2972, model for CR1 and CR3 adaptation	Sub-Cloning (next eight strains)	(4)
SMQ-1339	DGCC7710 (pNZ123), CmR	Control for ACR activity (Fig 3)	(5)
SMQ-1367	DGCC7710 (pNZAcrIIA2), CmR	Control for ACR activity (Fig 3)	This study
SMQ-1368	DGCC7710 (pNZAcrIIA4), CmR	Control for ACR activity (Fig 3)	This study
SMQ-1349	DGCC7710 (pNZAcr-4276), CmR	Control for ACR activity (Fig 3)	(3)
SMQ-1369	DGCC7710 (pNZAcr-1126), CmR	Control for ACR activity (Fig 3)	This study
SMQ-1370	DGCC7710 (pNZAcr-1811), CmR	Control for ACR activity (Fig 3)	This study
SMQ-1371	DGCC7710 (pNZAcr-1024), CmR	Control for ACR activity (Fig 3)	This study
SMQ-1372	DGCC7710 (pNZAcr-DT1), CmR	Control for ACR activity (Fig 3)	This study
SMQ-1335b	DGCC7710 w/CR1 spacer targeting 2972	Sub-Cloning (next eight strains)	(5)
SMQ-1350	SMQ-1335b (pNZ123), CmR	Control for ACR activity (Fig 3)	(3)
SMQ-1373	SMQ-1335b (pNZAcrIIA2), CmR	Test for ACR activity vs CR1 (Fig 3)	This study
SMQ-1374	SMQ-1335b (pNZAcrIIA4), CmR	Test for ACR activity vs CR1 (Fig 3)	This study
SMQ-1351	SMQ-1335b (pNZAcr-4276), CmR	Test for ACR activity vs CR1 (Fig 3)	(3)
SMQ-1375	SMQ-1335b (pNZAcr-1126), CmR	Test for ACR activity vs CR1 (Fig 3)	This study
SMQ-1376	SMQ-1335b (pNZAcr-1811), CmR	Test for ACR activity vs CR1 (Fig 3)	This study
SMQ-1377	SMQ-1335b (pNZAcr-1024), CmR	Test for ACR activity vs CR1 (Fig 3)	This study
SMQ-1378	SMQ-1335b (pNZAcr-DT1), CmR	Test for ACR activity vs CR1 (Fig 3)	This study
SMQ-1338	DGCC7710 w/CR3 spacer targeting 2972	Sub-Cloning (next eight strains)	(3)
SMQ-1352	SMQ-1338 (pNZ123), CmR	Control for ACR activity (Fig 3)	(3)
SMQ-1379	SMQ-1338 (pNZAcrIIA2), CmR	Test for ACR activity vs CR3 (Fig 3)	This study
SMQ-1380	SMQ-1338 (pNZAcrIIA4), CmR	Test for ACR activity vs CR3 (Fig 3)	This study
SMQ-1353	SMQ-1338 (pNZAcr-4276), CmR	Test for ACR activity vs CR3 (Fig 3)	(3)
SMQ-1381	SMQ-1338 (pNZAcr-1126), CmR	Test for ACR activity vs CR3 (Fig 3)	This study
SMQ-1382	SMQ-1338 (pNZAcr-1811), CmR	Test for ACR activity vs CR3 (Fig 3)	This study
SMQ-1383	SMQ-1338 (pNZAcr-1024), CmR	Test for ACR activity vs CR3 (Fig 3)	This study
SMQ-1384	SMQ-1338 (pNZAcr-DT1), CmR	Test for ACR activity vs CR3 (Fig 3)	This study
MG1363	*L. lactis,* host to phage p2	Sub-Cloning (next sixteen strains)	(6)
SMQ-1354	MG1363 (pNZ123, pL2Cas9), CmR, EmR	Control for SpCas9 activity (Fig 3)	(3)
SMQ-1385	MG1363 (pNZAcrIIA2, pL2Cas9), CmR, EmR	Control for SpCas9 activity (Fig 3)	This study
SMQ-1386	MG1363 (pNZAcrIIA4, pL2Cas9), CmR, EmR	Control for SpCas9 activity (Fig 3)	This study
SMQ-1355	MG1363 (pNZAcr-4276, pL2Cas9), CmR, EmR	Control for SpCas9 activity (Fig 3)	(3)
SMQ-1387	MG1363 (pNZAcr-1126, pL2Cas9), CmR, EmR	Control for SpCas9 activity (Fig 3)	This study
SMQ-1388	MG1363 (pNZAcr-1811, pL2Cas9), CmR, EmR	Control for SpCas9 activity (Fig 3)	This study
SMQ-1389	MG1363 (pNZAcr-1024, pL2Cas9), CmR, EmR	Control for SpCas9 activity (Fig 3)	This study
SMQ-1390	MG1363 (pNZAcr-DT1, pL2Cas9), CmR, EmR	Control for SpCas9 activity (Fig 3)	This study
SMQ-1356	MG1363 (pNZ123, pL2Cas9-44), CmR, EmR	Control for SpCas9 activity (Fig 3)	(3)
SMQ-1391	MG1363 (pNZAcrIIA2, pL2Cas9-44), CmR, EmR	Test for SpCas9 activity (Fig 3)	This study
SMQ-1392	MG1363 (pNZAcrIIA4, pL2Cas9-44), CmR, EmR	Test for SpCas9 activity (Fig 3)	This study
SMQ-1357	MG1363 (pNZAcr-4276, pL2Cas9-44), CmR, EmR	Test for SpCas9 activity (Fig 3)	(3)
SMQ-1393	MG1363 (pNZAcr-1126, pL2Cas9-44), CmR, EmR	Test for SpCas9 activity (Fig 3)	This study
SMQ-1394	MG1363 (pNZAcr-1811, pL2Cas9-44), CmR, EmR	Test for SpCas9 activity (Fig 3)	This study
SMQ-1395	MG1363 (pNZAcr-1024, pL2Cas9-44), CmR, EmR	Test for SpCas9 activity (Fig 3)	This study
SMQ-1396	MG1363 (pNZAcr-DT1, pL2Cas9-44), CmR, EmR	Test for SpCas9 activity (Fig 3)	This study
SMQ-1397	NEB5α (pDONR-AcrIIA6), KanR	Source of AcrIIA6 to suclone into pETG-20A	This study
SMQ-1398	Rosetta pLyS (pETG-20A-AcrIIA6), AmpR	Overexpression or AcrIIA6-D1811	This study

Phages	Description	Collection ID, GB Accession	Source
D5842	virulent *cos* -type phage of *S. thermophilus* DGCC7854, CRISPR-sensitive	GB: MH000602	(3)
D1024	virulent *cos* -type phage of *S. thermophilus* DGCC7854, CRISPR 'impeded adaptation'	GB: MH000603	(3)
D4276	virulent *cos* -type phage of *S. thermophilus* DGCC7854, CRISPR-resistant	GB: MF161328	(3)
D1811	virulent *cos* -type phage of *S. thermophilus* DGCC7854, CRISPR-resistant	GB: MH000604	This study
DT1	virulent *cos* -type phage of *S. thermophilus* SMQ-301	GB: AF085222	(7)
2972	virulent *pac* -type phage of *S. thermophilus* DGCC7710	GB: AY699705	(8)
D1126	virulent *pac* -type phage of *S. thermophilus* DGCC7710		This study
p2	virulent *cos* -type phage of *L. lactis* MG1363	GB: GQ979703	(9)
Plasmids	Description	Function	Source
pNZ123	Native vector, encodes chloramphenicol resistance	Negative control	(10)
AcrIIA2	pUC57 with AcrIIA2 synthesis construct, AcrIIA5 RBS	Template used to amplify AcrIIA2 for subcloning	BioBasic
AcrIIA4	pUC57 with AcrIIA4 synthesis construct, AcrIIA5 RBS	Template used to amplify AcrIIA4 for subcloning	BioBasic
pNZAcrIIA2	pNZ123 with AcrIIA2 insert in the XbaI cut site	anti-CRISPR AcrIIA2 expression	This study
pNZAcrIIA4	pNZ123 withAcrIIA4 insert in the XbaI cut site	anti-CRISPR AcrIIA4 expression	This study
pNZAcr-4276	pNZ123 with D4276_g28 insert in the XbaI cut site	anti-CRISPR AcrIIA6$_{D4276}$ expression	(3)
pNZAcr-1126	pNZ123 with AcrIIA5-D1126 insert in the XbaI cut site	anti-CRISPR AcrIIA6$_{D1126}$ expression	This study
pNZAcr-1811	pNZ123 with D1811_g26 insert in the XbaI cut site	anti-CRISPR AcrIIA6$_{D1811}$ expression	This study
pNZAcr-1024	pNZ123 with D1024_g29 insert in the XbaI cut site	anti-CRISPR AcrIIA6$_{D1024}$ expression	This study
pNZAcr-DT1	pNZ123 with DT1_g27 insert in the XbaI cut site	anti-CRISPR AcrIIA6$_{DT1}$ expression	This study
pL2Cas9	SpCas9 from pCas9 on a pTRKL2 backbone, for use in *L. lactis*	Negative control	(11)
pL2Cas9-44	pL2Cas9 with PS44 and PS_44_RC ligated into the BsaI cut site	Confer resistance against phage p2	(3)
pDONR201	Native vector, kanamycin resistance	Used to create the entry clone pDONR-AcrIIA6	Invitrogen
pDONR-AcrIIA6	pDONR entry clone with D1811_g26 insert	Used to subclone into pETG-20A	This study
pETG-20A	Native expression vector, ampicilin resistance	Used to create the expression clone pETG-20A-AcrIIA6	EMBL
pETG-20A-AcrIIA6	pETG-20A expression vector with D1811_g26 insert	anti-CRISPR AcrIIA6$_{D1811}$ overexpression	This study
Oligos	Sequence 5’-3’	Function	Source
Yc70	TGCTGAGACAACCTAGTCTCTC	CR1 locus screening (DGCC7854)	(12)
CR1-rev	TAAACAGAGCCTCCCTATCC	CR1 locus screening (DGCC7854)	(12)
pNZins_F	AATGTCACTAACCTGCCCCG	pNZ123 insert screening	(5)
pNZins_R	CATTGAACATGCTGAAGAGC	pNZ123 insert screening	(5)
GibsonUp_F	ATTACAGCTCCAGATCCAGTACTGAATTCT	pNZAcrIIA2 and pNZAcrIIA4 constructions	This study
GibsonDown_R	GAAAATATGCACTCGAGAAGCTTGAGCTCT	pNZAcrIIA2 and pNZAcrIIA4 constructions	This study
D1126_ACR_F	ATTACAGCTCCAGATCCAGTACTGAATTCTTCTGAAAAAGTTTGGGAAGTAGCT	pNZAcr-1126 construction	This study
D1126_ACR_R	GAAAATATGCACTCGAGAAGCTTGAGCTCTACTAACACCAGTTTGTCTTTCTAAAT	pNZAcr-1126 construction	This study
D1811_g26_F	ATTACAGCTCCAGATCCAGTACTGAATTCTTCGCTGAAAAAGTTTGGGAAGT	pNZAcr-1811 and pNZAcr-1024 constructions	This study
D1811_g26_R	GAAAATATGCACTCGAGAAGCTTGAGCTCTCCTCTCTCTTATGATAGTCTGCCA	pNZAcr-1811 and pNZAcr-1024 constructions	This study
DT1_g27_F	ATTACAGCTCCAGATCCAGTACTGAATTCTACATCGCTGAAAACGTTTGGA	pNZAcr-DT1 construction	This study
DT1_g27_R	GAAAATATGCACTCGAGAAGCTTGAGCTCTTGTATCCATTGTTTTTACCTCGTTT	pNZAcr-DT1 construction	This study
GWF_AcrIIA6	GGGGACAAGTTTGTACAAAAAAGCAGGCTTAGAAAACCTGTACTTCCAGGGTAAAATAAATGACGACATCAA	pDONR-1811 construction and screening	This study
GWR_AcrIIA6	GGGGACCACTTTGTACAAGAAAGCTGGGTCTTATTATCGAATGTTTTCGAGAT	pDONR-1811 construction and screening	This study
sequence	Description	GB accession	Source
AcrIIA5-D1126	RBS region: TAATTAAAAAAAGAAAGAAGGAAGTAAAA + D1126_AcrIIA5	D1126_AcrIIA5 GB: MH000605	This study
AcrIIA2	AcrIIA5 RBS region: TTAAAAAAAGAAAGAAGGAAGTAAAA + AcrIIA2	AcrIIA2 GB: CP002001.1 locus_tag: LMOG_03147	(13)
AcrIIA4	AcrIIA5 RBS region: TTAAAAAAAGAAAGAAGGAAGTAAAA + AcrIIA4 codon optimized (residue R33 CGC → CGA)	AcrIIA4 GB : CP002001.1 locus_tag: LMOG_02993 [https://www.ncbi.nlm.nih.gov/nuccore/CP002001.1]	(13)

underline : Extensions used for Gibson or Gateway cloning

Supplementary Table 3. SpCas9 and St1Cas9 spacer (guide) sequences.

Target	Sequence 5’-3’
SpCas9 *EMX1*	GAGTCCGAGCAGAAGAAGAA
SpCas9 *FANCF*	GGAATCCCTTCTGCAGCACC
SpCas9 *RUNX1*	GCATTTTCAGGAGGAAGCGA
St1Cas9 *EMX1*	GAAGGGCCTGAGTCCGAGCA
St1Cas9 *FANCF*	GTAGGTAGTGCTTGAGACCG
St1Cas9 *RUNX1*	GAGGTATCCAGCAGAGGGGA

Supplementary Table 4. AcrIIA Gblocks.

Gblock	Sequence 5'-3'
AcrIIA2[24]	GAATTCC*TGTACA*AGAAAAGGCCGGCGGCCACGAAAAAGGCCGGCCAG<br>GCAAAAAAGAAAAAGGGCGGAGGCTCTGGCGGCGGAAGC*GCGGCCGC*<br>CATGACGCTGACCCGCGCTCAGAAAAAATACGCCGAGGCGATGCATG<br>AGTTTATCAATATGGTTGATGACTTTGAAGAATCAACGCCTGACTTTGC<br>AAAAGAGGTTCTGCACGACTCCGACTATGTGGTCATTACAAAAAACGA<br>GAAATATGCCGTGGCACTCTGTAGTCTCTCCACAGATGAATGTGAGTA<br>CGATACTAACTTGTATTTGGATGAAAAGCTCGTCGATTACAGCACAGT<br>TGATGTCAACGGAGTGACATATTACATCAATATAGTGGAAACAAATGA<br>CATAGATGATCTTGAAATTGCGACCGACGAGGACGAGATGAAGTCTGG<br>AAACCAAGAGATTATTCTTAAGTCCGAACTGAAGTGA[*]TAA[*]CTCGAGT<br>CTAGACGTTTAAACCCTGCAGGCTGTG
AcrIIA4[24]	GAATTCC*TGTACA*AGAAAAGGCCGGCGGCCACGAAAAAGGCCGGCCAG<br>GCAAAAAAGAAAAAGGGCGGAGGCTCTGGCGGCGGAAGC*GCGGCCGC*<br>CATGAACATTAACGACCTCATACGAGAGATTAAGAACAAAGATTACAC<br>CGTCAAACTGTCAGGAACTGATAGTAACTCAATCACCCAGCTTATTAT<br>CAGGGTAAACAATGATGGGAATGAATATGTGATATCTGAGAGCGAAA<br>ACGAGTCTATCGTCGAGAAATTCATTTCCGCTTTTAAGAACGGGTGGA<br>ATCAGGAATATGAGGATGAAGAAGAATTTTACAATGACATGCAGACG<br>ATCACGTTGAAAAGTGAACTGAACTGA[*]TAA[*]CTCGAGTCTAGACGTTT<br>AAACCCTGCAGGCTGTG
AcrIIA5 D1126	GAATTCC*TGTACA*AGAAAAGGCCGGCGGCCACGAAAAAGGCCGGCCAG<br>GCAAAAAAGAAAAAGGGCGGAGGCTCTGGCGGCGGAAGC*GCGGCCGC*<br>CATGGCCTATGGTAAAAGTAGGTATAACTCCTATCGCAAACGCAACTT<br>CAGCATCTCTGACAATCAAAGGAGAGAATACGCAAAGAAAATGAAGG<br>AACTTGAACAGGCATTTGAGAACCTCGACGGATGGTACTTGAGTAGCA<br>TGAAAGATTCTGCGTATAAGGATTTCGGTAAGTACGAAATCCGACTTT<br>CAAATCACTCAGCCGACAATCGCTATCATGACCTGGAGAACGGCCGCT<br>TGATCGTGAATGTGAAAGCAAGCAAACTTAACTTTGTCGATATTATCG<br>AGAATAAACTCGGCAAGATCATCGAAAAAATTGATACCCTGGACCTTG<br>ATAAATATCGCTTCATCAATGCGACCAAGCTGGAGAGGGACATTAAGT<br>GCTACTATAAAGGGTATAAGACTAAGAAAGACGTTATCTGA[*]TAA[*]CTC<br>GAGTCTAGACGTTTAAACCCTGCAGGCTGTG
AcrIIA5 D4276	GAATTCC*TGTACA*AGAAAAGGCCGGCGGCCACGAAAAAGGCCGGCCAG<br>GCAAAAAAGAAAAAGGGCGGAGGCTCTGGCGGCGGAAGC*GCGGCCGC*<br>CATGGCCTATGGTAAAAGTAGGTATAACTCCTATCGCAAACGCTCTTTC<br>AACAGGTCTAACAAACAAAGGAGAGAATACGCACAGGAAATGGACCG<br>CCTTGAAAAGGCATTTGAGAACCTCGACGGATGGTACTTGAGTAGCAT<br>GAAAGATTCTGCGTATAAGGATTTCGGTAAGTACGAAATCCGACTTTC<br>AAATCACTCAGCCGACAATAAATATCATGACCTGGAGAACGGCCGCTT<br>GATCGTGAATATCAAAGCAAGCAAACTTAACTTTGTCGATATTATCGA<br>GAATAAACTCGACAAGATCATCGAAAAAATTGATAAGCTGGACCTTGA<br>TAAATATCGCTTCATCAATGCGACCAACCTGGAGCACGACATTAAGTG<br>CTACTATAAAGGGTTTAAGACTAAGAAAGAGGTTATCTGA[*]TAA[*]CTCG<br>AGTCTAGACGTTTAAACCCTGCAGGCTGTG
AcrIIA6 D1024	GAATTCC*TGTACA*AGAAAAGGCCGGCGGCCACGAAAAAGGCCGGCCAG<br>GCAAAAAAGAAAAAGGGCGGAGGCTCTGGCGGCGGAAGC*GCGGCCGC*<br>CATGAAGATCAACGACGACATAAAGGAATTGATCCTCGAATACATGTC<br>CCGATATTTTAAATTCGAGAATGACTTCTATAAACTGCCGGGAATCAA

	ATTTACCGATGCTAACTGGCAGAAGTTCAAAAATGGGGGACTGATAT<br>CGAGAAGATGGGGGCGGCCAGGGTGAATGCGATGCTTTCATGTCTCTT<br>CGAAGACTTCGAGTTGGCAATGATTGGAAAAGCCCAAACTAACTATTA<br>TATTGACAATTCTCTCAAGCTTAATATGCCATTTTACGCTTATTACGAC<br>ATGTTTAAAAAGCAGTTGCTTATTAACTGGCTGAAAAATAATCGCGAC<br>GACGTTATTTGCGGCACGGGTAGGATGTATACTGCGTCAGGGAACTAT<br>ATTGCAAACGCGTACCTGGAGGTGGCTCTGGAGTCTAGCCGACTGGGC<br>GGTGGAGAGTATATGCTTCAGATGCGCTTCAAAAATTATAGTCGCAGT<br>CAAGAGCCTATCCCATCAGGACGACAGAACCGACTTGAATGGATCGAA<br>AACAATCTTGAGAACATCCGGTGA*TAA*CTCGAGTCTAGACGTTTAAA<br>CCCTGCAGGCTGTG
AcrIIA6 D1811	GAATTCC*TGTACA*AGAAAAGGCCGGCGGCCACGAAAAAGGCCGGCCAG<br>GCAAAAAAGAAAAAGGGCGGAGGCTCTGGCGGCGGAAGC*GCGGCCGC*<br>CATGAAGATCAACGACGACATAAAGGAATTGATCCTCGAATACATGTC<br>CCGATATTTTAAATTCGAGAATGACTTCTATAAACTGCCGGGAATCAA<br>ATTTACCGATGCTAACTGGCAGAAGTTCAAAAATGGGGGACTGATAT<br>CGAGAAGATGGGGGCGGCCAGGGTGAATGCGATGCTTGATTGTCTCTT<br>CGATGACTTCGAGTTGGCAATGATTGGAAAAGCCCAAACTAACTATTA<br>TAATGACAATTCTCTCAAGATGAATATGCCATTTTACACTTATTACGAC<br>ATGTTTAAAAAGCAGCAACTTCTCAAGTGGCTGAAAAATAATCGCGAC<br>GACGTTATTGGCGGCACGGGTAGGATGTATACTGCGTCAGGGAACTAT<br>ATTGCAAACGCGTACCTGGAGGTGGCTCTGGAGTCTAGCTCACTGGGC<br>TCAGGAAGCTATATGCTTCAGATGCGCTTCAAAGATTATAGTAAAGGA<br>CAAGAGCCTATCCCATCAGGACGACAGAACCGACTTGAATGGATCGAA<br>AACAATCTTGAGAACATCCGGTGA*TAA*CTCGAGTCTAGACGTTTAAA<br>CCCTGCAGGCTGTG
AcrIIA6 DT1	GAATTCC*TGTACA*AGAAAAGGCCGGCGGCCACGAAAAAGGCCGGCCAG<br>GCAAAAAAGAAAAAGGGCGGAGGCTCTGGCGGCGGAAGC*GCGGCCGC*<br>CATGAAGATCAACAACGACATAAAGGAATTGATCCTCGAATACGTGTC<br>CCGATATTTTAAATTCGAGAATGACTTCTATAAACTGCCGGGAATCAA<br>ATTTACCGATGCTAACTGGCAGAAGTTCAAAAATGGGGAAACTTCCAT<br>CGAGAAGATGGGGGCGGCCAGGGTGAATGCGATGCTTTCATGTCTCTT<br>CGAAGACTTCGAGTTGGCAATGATTGGAAAAGCCCAAACTAACTATTA<br>TATTGACAATTCTCTCAAGCTTAATATGCCATTTTACGCTTATTACGAC<br>ATGTTTAAAAAGCAGTTGCTTATTAACTGGCTGAAAAATAATCGCGAC<br>GACGTTATTGGCGGCACGGGTAGGATGTATACTGCGTCAGGGAACTAT<br>ATTGCAAACGCGTACCTGGAGGTGGCTCTGGAGTCTAGCTCACTGGGC<br>TCAGGAAGCTATATGATACAGATGCGCTTCAAAGATTATAGTAAAGGA<br>CAAGAGCCTATCCCATCAGGACGAAAGAACCGACTTGAATGGATCGA<br>AAACAATCTTGAGAACATCCGGTGA*TAA*CTCGAGTCTAGACGTTTAA<br>ACCCTGCAGGCTGTG

Restriction sites: *BsrGI* (*TGTACA*) to make mScarlet-I fusions; *NotI* (*GCGGCCGC*) for untagged constructs

Nucleophosmin NLS

$(GGGS)_2$ linker

AcrIIA ORF

TGA*TAA* Stop codons

Supplementary Table 5. PCR primers used for Surveyor assays.

Target	Primer
EMX1 Forward	CCATCCCCTTCTGTGAATGT
EMX1 Reverse	GGAGATTGGAGACACGGAGA
FANCF Forward	GGGCCGGGAAAGAGTTGCTG
FANCF Reverse	GCCCTACATCTGCTCTCCCTCC
RUNX1 Forward	CCAGCACAACTTACTCGCACTTGAC
RUNX1 Reverse	CATCACCAACCCACAGCCAAGG

Supplementary References

1. Kearse, M. *et al.* Geneious Basic : an integrated and extendable desktop software platform for the organization and analysis of sequence data. *Bioinformatics* **28**(12), 1647-1649 (2012).

2. Gouet, P., Courcelle, E., Stuart, D. I. & Metoz, F. ESPript: analysis of multiple sequence alignments in PostScript. *Bioinformatics* **15,** 305–308 (1999).

3. Hynes, A. P. *et al.* An anti-CRISPR from a virulent streptococcal phage inhibits *Streptococcus pyogenes* Cas9. *Nat Microbiol* (2017). doi:10.1038/s41564-017-0004-7

4. Barrangou, R. *et al.* CRISPR provides acquired resistance against viruses in prokaryotes. *Science* **315,** 1709–1712 (2007).

5. Hynes, A. P., Labrie, S. J. & Moineau, S. Programming native CRISPR arrays for the generation of targeted immunity. *MBio* **7,** e00202-16 (2016).

6. Gasson, M. J. Plasmid complements of *Streptococcus lactis* NCDO 712 and other lactic streptococci after protoplast-induced curing. *J Bacteriol* **154,** 1–9 (1983).

7. Tremblay, D. M. & Moineau, S. Complete genomic sequence of the lytic bacteriophage DT1 of *Streptococcus thermophilus*. *Virology* **255,** 63–76 (1999).

8. Lévesque, C. *et al.* Genomic organization and molecular analysis of virulent bacteriophage 2972 infecting an exopolysaccharide-producing *Streptococcus thermophilus* strain. *Appl Env. Microbiol* **71,** 4057–4068 (2005).

9. Higgins, D. L., Sanozky-Dawes, R. B. & Klaenhammer, T. R. Restriction and modification activities from *Streptococcus lactis* ME2 are encoded by a self-transmissible plasmid,

pTN20, that forms cointegrates during mobilization of lactose-fermenting ability. *J Bacteriol* **170,** 3435–3442 (1988).

10. De Vos, W. M. Gene cloning and expression in lactic streptococci. *FEMS Microbiol Lett* **46,** 281–295 (1987).

11. Lemay, M.-L., Tremblay, D. M. & Moineau, S. Genome engineering of virulent lactococcal phages using CRISPR-Cas9. *ACS Synth Biol* (2017). doi:10.1021/acssynbio.6b00388

12. Horvath, P. *et al.* Diversity, activity, and evolution of CRISPR loci in *Streptococcus thermophilus*. *J Bacteriol* **190,** 1401–1412 (2008).

13. Rauch, B. J. *et al.* Inhibition of CRISPR-Cas9 with bacteriophage proteins. *Cell* **168,** 150–158.e10 (2017).
